# Histone deacetylase 3 promotes innate antiviral immunity through deacetylation of TBK1

**DOI:** 10.1007/s13238-020-00751-5

**Published:** 2020-08-09

**Authors:** Jie-lin Tang, Qi Yang, Chong-hui Xu, He Zhao, Ya-ling Liu, Can-yu Liu, Yuan Zhou, Dong-wei Gai, Rong-juan Pei, Yun Wang, Xue Hu, Bo Zhong, Yan-yi Wang, Xin-wen Chen, Ji-zheng Chen

**Affiliations:** 1 State Key Laboratory of Virology, Wuhan Institute of Virology, Center for Biosafety Mega-Science, Chinese Academy of Sciences, Wuhan, 430071 China; 2 University of Chinese Academy of Sciences, Beijing, 100049 China; 3 Medical Research Institute, School of Medicine, Wuhan University, Wuhan, 430071 China; 4 Guangzhou Institutes of Biomedicine and Health, Chinese Academy of Sciences, Guangzhou, 510530 China

**Keywords:** TBK1, HDAC3, deacetylation, IRF3, innate immune

## Abstract

TANK-binding kinase 1 (TBK1), a core kinase of antiviral pathways, activates the production of interferons (IFNs). It has been reported that deacetylation activates TBK1; however, the precise mechanism still remains to be uncovered. We show here that during the early stage of viral infection, the acetylation of TBK1 was increased, and the acetylation of TBK1 at Lys241 enhanced the recruitment of IRF3 to TBK1. HDAC3 directly deacetylated TBK1 at Lys241 and Lys692, which resulted in the activation of TBK1. Deacetylation at Lys241 and Lys692 was critical for the kinase activity and dimerization of TBK1 respectively. Using knockout cell lines and transgenic mice, we confirmed that a HDAC3 null mutant exhibited enhanced susceptibility to viral challenge via impaired production of type I IFNs. Furthermore, activated TBK1 phosphorylated HDAC3, which promoted the deacetylation activity of HDAC3 and formed a feedback loop. In this study, we illustrated the roles the acetylated and deacetylated forms of TBK1 play in antiviral innate responses and clarified the post-translational modulations involved in the interaction between TBK1 and HDAC3.

**Electronic supplementary material:**

The online version of this article (10.1007/s13238-020-00751-5) contains supplementary material, which is available to authorized users.

## Introduction

The detection of pathogen-associated molecular patterns (PAMPs) on microbial pathogens via pattern-recognition receptors (PRRs) is the first step in eliciting innate immune responses in the host. The activation of PRRs triggers downstream signaling cascades that lead to the rapid production of type I interferons (IFNs) and proinflammatory cytokines that eliminate pathogens (Akira et al., 2006; Medzhitov, 2007; Takeuchi and Akira, 2010). Post-translational modifications (PTMs) act as vital regulators of cellular signal transduction systems during innate immune responses. Lysine acetylation, which affects the stability, activation, and subcellular localization of proteins, as well as the interaction of proteins with other proteins or DNA, has been identified as a critical regulator of the innate immune system (Mowen and David, 2014; Li et al., 2016; Dai et al., 2019). Several non-histone signal transducing proteins, which are important for TLR pathways, and transcriptional regulators involved in the expression of inflammatory cytokines are regulated by acetylation (Cao et al., 2008). However, the crosstalk that occurs between acetylation, conventional PTMs, and modulation of the kinase activity of signaling transducers in the innate immune response is still unknown.

Interferon regulatory factor 3 (IRF3) acts as a master transcription factor responsible for the production of IFN-β (IFN-β) and is essential for the establishment of innate immunity (Honda and Taniguchi, 2006). TBK1 is a key kinase for IRF3 activation that leads to the expression of type I IFNs (Fitzgerald et al., 2003). Several positive or negative regulatory mechanisms for the control of TBK1-mediated antiviral responses have been described (An et al., 2015; Song et al., 2016; Liu et al., 2017). The activation and function of TBK1 are orchestrated by ubiquitination, dimerization, and phosphorylation (Li et al., 2011; Tu et al., 2013). Seven lysine residues on TBK1 have been shown to be modified by acetylation, and Lys241 deacetylation is critical for the kinase activity of TBK1 (Li et al., 2016). However, the precise mechanism still remains to be uncovered.

Epigenetic regulation is important for innate immune responses, especially for the modulation of signaling regulators, the transcription of genes encoding inflammatory products, and PTMs required for the activation of signaling pathways (Smale et al., 2014; Li et al., 2016). HDAC3, which belongs to the Class I HDACs, is thought to be a classical histone deacetylase, and non-histone targets have been reported (Mihaylova et al., 2011). Although the regulation of HDAC1, 4, 6, 8 or 9 in innate immunity has been revealed (Li et al., 2016; Meng et al., 2016; Yang et al., 2018), little is unknown about the role HDAC3 plays in anti-microbial innate immunity. Moreover, the phosphorylation of HDAC3 promotes its deacetylase activity, but the kinase for HDAC3 is unknown (Zhang et al., 2005).

Here, we report that HDAC3 acts as a positive regulator in antiviral immune responses. HDAC3 deficiency suppressed the production of IFN-β, a type I IFN, both *in vitro* and *in vivo*. HDAC3 deacetylated TBK1 to promote the dimerization and activation of TBK1. The acetylation of TBK1 at Lys241 during the early stage of viral infection enhanced the recruitment of IRF3 to TBK1. Furthermore, activated TBK1 further phosphorylated HDAC3 to enhance the activity of deacetylated HDAC3 and activate TBK1. Our results provide insights into the PTMs involved in the interaction between TBK1 and HDAC3 during antiviral innate responses.

## Results

### HDAC3 positively regulates type I IFN production

**Figure 1:**
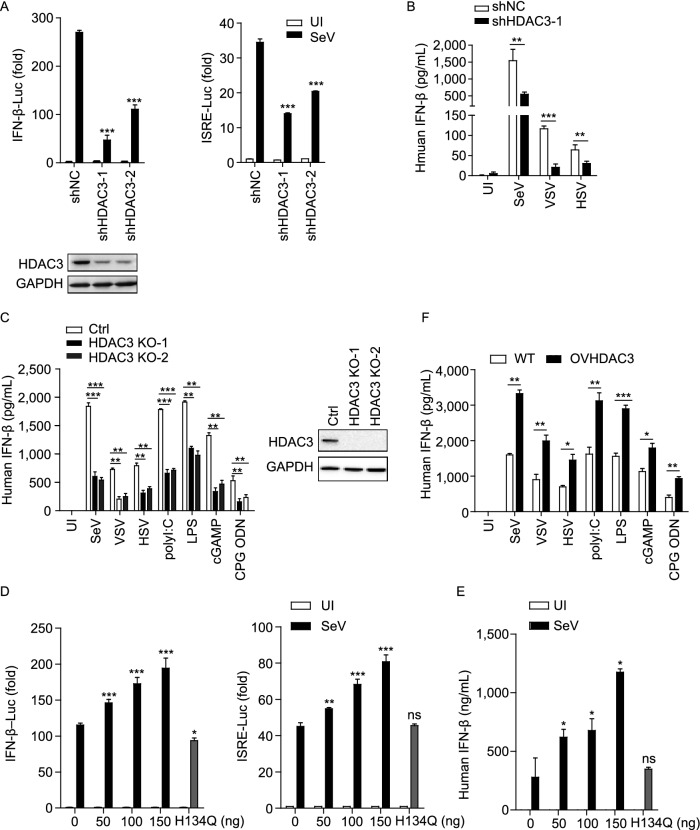
**HDAC3 positively regulates type I interferon production**. (A) Luciferase activity in shNC, shHDAC3-1 or shHDAC3-2 cell lines (1 × 10^5^), transfected for 36 h with a luciferase reporter for IFN-β (IFN-β-luc) or for ISRE (ISRE-luc), uninfected or infected with SeV for 10 h before luciferase assays were performed. HDAC3-Knockdown efficiency in the shHDAC3 cell lines were confirmed by immunoblotting analysis (lower). (B) ELISA analysis of IFN-β in the supernatant of shNC, shHDAC3-1 cell lines, followed by infection with or without SeV, VSV, HSV-1. (C) ELISA of IFN-β in the supernatant of THP-1 HDAC3-deficient cells (HDAC3 KO-1, HDAC3 KO-2), followed by infection with different stimulus (as shown). HDAC3 knockout efficiency in the THP-1 cells were confirmed by immunoblotting analysis (right). (D) Luciferase activity assay in HEK293T cells (1 × 10^5^), co-transfected for 36 h with empty vectors, increased amounts of HDAC3 expression plasmids or Flag tagged HDAC3-H134Q (100 ng) together with a luciferase reporter for IFN-β (IFN-β-luc) or for ISRE (ISRE-luc), followed by infection with or without SeV for 10 h before luciferase assays were performed. (E) ELISA analysis of IFN-β in the supernatant of HEK293T cells (1 × 10^5^), transfected for 36 h with empty vectors, increased amounts of HDAC3 expression plasmids or Flag tagged HDAC3-H134Q (100 ng), followed by infection with or without SeV for 10 h. (F) ELISA of IFN-β analysis in the supernatant of HDAC3-stable-expressing THP-1 cells, followed by infection with different stimulus (as shown). Data are representative of three independent experiments. Graphs show mean ± SD; *n* = 3. *P < 0.05; ** P < 0.01; ***P < 0.001 (Student’s *t*-test)

In a shRNA library screen, we found that knockdown of HDAC3 impaired Sendai virus (SeV)-induced activation via the *IFN-β* (IFNB1) gene promoter. To verify this phenomenon, two shRNA-mediated knockdown of HDAC3 was performed in HEK293T cells. HDAC3 knockdown reduced the promoter activity of IFN-β and IFN-stimulated response elements (ISRE) (Fig. 1A). In addition, after stimulation with various inducers, including SeV, vesicular stomatitis virus (VSV), and herpes simplex virus (HSV-1), IFNB1 production in the HDAC3-knockdown samples was significantly lower compared to the control samples (Fig. 1B). Similarly, knockdown of HDAC3 using siRNA validated these results (Fig. S1A and S1B). We further generated HDAC3-deficient THP-1 cells by CRISPR/Cas9 in order to investigate whether HDAC3 was required for virus-induced type I IFN responses in immunocytes. The secretion of IFN-β from HDAC3-deficient THP-1 cells was impaired as compared with their secretion from the control cells after stimulation by various inducers, including RNA viruses (SeV and VSV), DNA virus (HSV), synthetic RNA duplex poly (I:C), bacterial lipopolysaccharide (LPS), cGAMP, or CpG oligodeoxynucleotide (ODN) (Fig. 1C). We obtained similar results *in vitro* using a macrophage cell line (RAW264.7) with HDAC3 knockdown (Fig. S1C). These results indicated specific involvement of HDAC3 in the expression of type I IFNs.

In order to further confirm the contribution of HDAC3 to antiviral responses, we stimulated HDAC3-overexpressing HEK293T cells with SeV. The promoter activity of IFNB1 or ISRE and the production of IFN-β were significantly higher when HDAC3 was overexpressed as compared to samples transfected with the empty vector rather than HDAC3 deacetylase inactive mutant (H134Q), and the agonists occurred in a dose-dependent manner after infection with SeV (Fig. 1D and 1E). Accordingly, the production of IFN-β was higher in HDAC3-overexpressing THP-1 cells stimulated with various inducers (Fig. 1F). Together these results suggested a positive role for HDAC3 in PRR-induced induction of type I IFN in various cell types.

### Pivotal role for HDAC3 in the antiviral response *in vivo*

**Figure 2:**
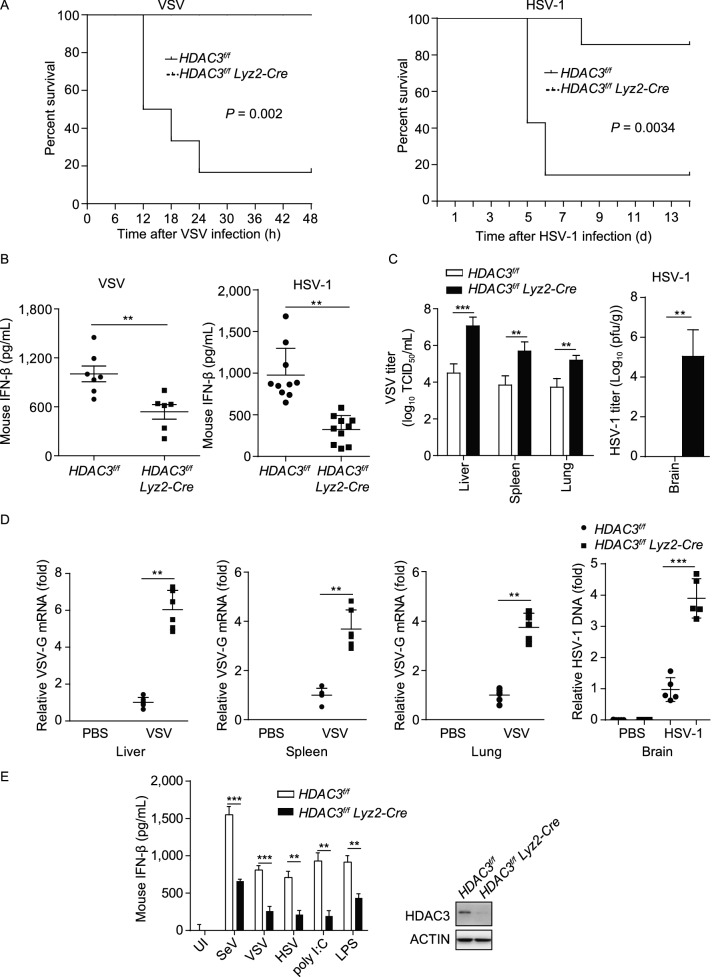
**Pivotal role for HDAC3 in the antiviral response** ***in vivo***. (A) Survival (Kaplan-Meier curve) of 8-week-olds *HDAC3*^*fl*/*fl*^ (*n* = 5, 7) and *HDAC3*^*fl*/*fl*^ *Lyz2-Cre* mice (*n* = 5, 7) intraperitoneal injected with VSV-GFP or HSV-1-GFP (7 ×10^7^ PFU/g, 2 × 10^7^ PFU/g) [plaque-forming unit] monitored for 48 h or 2 w. (B) ELISA analysis of IFN-β in the sera from *HDAC3*^*fl*/*fl*^ (*n* = 7, 10) and *HDAC3*^*fl*/*fl*^ *Lyz2-Cre* mice (*n* = 6, 10) after VSV or HSV-1 infection. (C) VSV load in the liver, spleen, lungs or HSV load in the brain of *HDAC3*^*fl*/*fl*^ or *HDAC3*^*fl*/*fl*^ *Lyz2-Cre* mice (*n* = 5 per group) after intraperitoneal infection with VSV (24 h) (as in left) or HSV-1 (5 days) (as in A), assessed by endpoint-dilution assay and presented as 50% tissue culture infectious dose (TCID_50_). (D) Quantitative PCR analysis of VSV RNA in the liver, spleen, lungs or HSV-1 DNA in the brain of *HDAC3*^*fl*/*fl*^ or *HDAC3*^*fl*/*fl*^ *Lyz2-Cre* mice after intraperitoneal injection of PBS or VSV (24 h) or HSV-1 (5 d) (as in A). (E) ELISA analysis of IFN-β (left) in the supernatant of *HDAC3*^*fl*/*fl*^ or *HDAC3*^*fl*/*fl*^ *Lyz2-Cre* bone marrow derived macrophages (BMDMs), infected with SeV, VSV, HSV-1, poly (I:C) and LPS. HDAC3-deficiency in BMDMs were confirmed by immunoblotting analysis (right). Data are representative of three independent experiments. Graphs show mean ± SD; **P < 0.01; ***P < 0.001 (Student’s *t*-test)

To further elucidate the importance of HDAC3 in antiviral immunity *in vivo*, we adopted a strategy to generate *HDAC3 flox*/*flox-Lyz2-Cre* mice (*HDAC3*^*fl*/*fl*^ *Lyz2-Cre*), which undergo deletion of loxP-flanked HDAC3 alleles (*HDAC3*^*fl*/*fl*^) specifically in myeloid cells via Cre recombinase expressed from the myeloid cell-specific gene *Lyz2* (*Lyz2-Cre*). We then challenged these mice with VSV or HSV and found that the mortality rate of *HDAC3*^*fl*/*fl*^ *Lyz2-Cre* was greater than that of the *HDAC3*^*fl*/*fl*^ littermates (Fig. 2A). HDAC3-deficient mice produced decreased levels of serum IFN-β in response to infection with VSV or HSV than their *HDAC3*^*fl*/*fl*^ littermates (Fig. 2B). The VSV titers and replication in various organs and HSV titers and replication in the brain were also significantly greater in HDAC3-deficient mice than in their *HDAC3*^*fl*/*fl*^ counterparts (Fig. 2C and 2D). The secretion of IFN-β from HDAC3-deficient bone marrow-derived macrophages (BMDMs) from *HDAC3*^*fl*/*fl*^ *Lyz2-Cre* mice was severely impaired as compared with the secretion from *HDAC3*^*fl*/*fl*^ BMDMs stimulated by various inducers, including SeV, VSV, HSV-1, poly (I:C), or (LPS) (Fig. 2E). Therefore, HDAC3 was required for the efficient production of type I IFNs and for the resistance of these mice to viral infection.

### Deficiency in HDAC3 impairs virus-induced phosphorylation of IRF3

**Figure 3:**
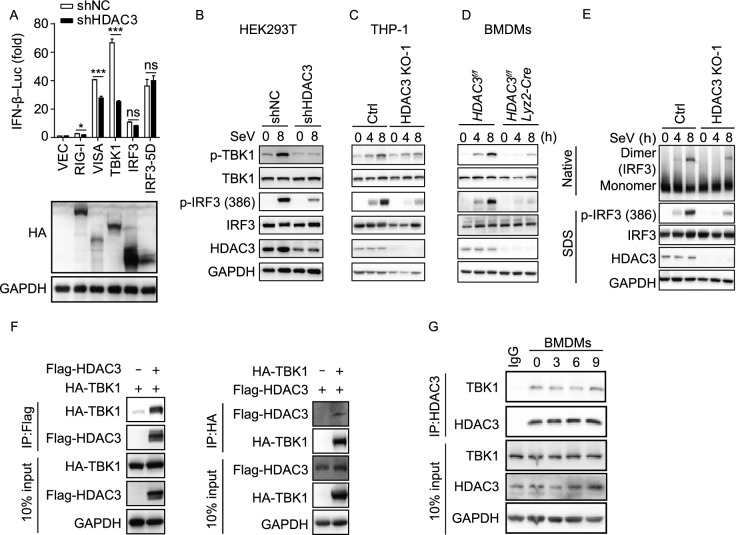
**Deficiency of HDAC3 impairs phosphorylation of IRF3 by TBK1**. (A) Luciferase activity (upper) in shNC or shHDAC3 cell lines (1 × 10^5^), transfected with an IFN-β-Luc, together with control vector (VEC), HA-tagged RIG-I, VISA, TBK1, IRF3, or IRF3-5D; the protein expression of RIG-I, VISA, TBK1, IRF3, or IRF3-5D were confirmed by immunoblotting analysis (lower). (B–D) Immunoblot analysis of phosphorylated and total TBK1 or IRF3 in shHDAC3 HEK293T cell lines (B), THP-1 HDAC3 deficient cell lines (C), *HDAC3*^*fl*/*fl*^ or *HDAC3*^*fl*/*fl*^ *Lyz2-Cre* BMDMs (D), followed by infection with SeV for the indicated times. (E) Immunoblot analysis of IRF3 in dimerization in HDAC3-deficient THP-1 cells and left infected with SeV for the indicated times, followed by native PAGE. (F) The interaction between HDAC3 and TBK1. HEK293T cells (1.5 × 10^6^) were co-transfected with the indicated plasmids (3 μg each) for 48 h. Co-immunoprecipitation and immunoblotting were performed with the indicated antibodies. (G) Endogenous HDAC3 dynamically interacts with TBK1, followed by infection with SeV. BMDMs were left uninfected or infected with SeV for the indicated times. Co-immunoprecipitation and immunoblotting were performed with the indicated antibodies. Data are representative of three independent experiments. Graphs show mean ± SD; *n* = 3. ns, no significant differences;*P < 0.05; ***P < 0.001 (Student’s *t*-test)

In order to understand the mechanism by which HDAC3 regulates innate antiviral signaling, we screened the signaling pathways responsible for initiating the production of IFN-β. We investigated if HDAC3 influenced the levels of PRR-triggered signaling molecules using luciferase reporter assays. Knockdown of HDAC3 significantly inhibited IFNB1 reporter activity induced by upstream activators, including RIG-I, VISA, and TBK1, but not IRF3 or IRF3-5D (Fig. 3A). Phosphorylation of the transcription factor IRF3 at Ser386 has been shown to result in oligomerization, which has been strongly correlated with its full activation (Suhara et al., 2000). We found reduced phosphorylation of IRF3 at Ser386 and TBK1 at Ser172 induced by SeV in HDAC3-knockdown HEK293T cells, HDAC3-deficient THP-1 cells, or HDAC3-deficient BMDMs (*HDAC3*^*fl*/*fl*^ *Lyz2-Cre*) than in their control counterparts (Fig. 3B–D). Similarly, dimerization of IRF3 was weakened greatly in HDAC3-deficient THP-1 cells infected with SeV as compared to control THP-1 cells (Fig. 3E). Simultaneously, the results of immunoprecipitation showed that the interaction of TBK1 with VISA, TRAF6, or TANK had minimal enhancement when HDAC3 was present (Fig. S2A–C). These data indicated that HDAC3 might be involved in the IRF3-mediated innate immune response by targeting TBK1 and affecting the activation of IRF3.

In order to investigate whether TBK1 was able to interact with HDAC3, we overexpressed HA-tagged TBK1 and Flag-tagged HDAC3 together in HEK293T cells. Immunoprecipitation and immunoblot assays indicated that HDAC3 and TBK1 associated together (Fig. 3F). The immunofluorescence detection showed co-localization of HDAC3 and TBK1 in HEK293T cells (Fig. S3A). Endogenous IP analysis suggested that HDAC3 constitutively interacted with TBK1 during SeV infection (Fig. 3G). It has been reported and our experiments revealed that HDAC3 shuttles between the nucleus and cytoplasm (Fig. S3B) (Chini et al., 2010). We constructed HDAC3 mutants that predominantly localize to cytoplasm (HDAC3-ΔNLS) and nucleus (2×NLS-HDAC3), respectively (Fig. S3C) (Park et al., 2014). As expected, the phosphorylation of TBK1 and IRF3 was enhanced with the expression of wild-type HDAC3 or cytoplasmic HDAC3 (HDAC3-ΔNLS), but not the nuclear HDAC3 (Fig. S3D). The HDAC3-△NLS, not HDAC3-NLS, could promote the IFN-beta mRNA level and the production of IFN-beta during SeV challenge, indicating that the cytoplasmic HDAC3 that promotes activation of IRF3 (Fig. S3E and S3F). These results implied that regulation HDAC3 gene transcription had little effect on activation of IRF3. Collectively, these data suggested that HDAC3 could interact with TBK1 to promote the phosphorylation and activation of IRF3 by TBK1.

### HDAC3 directly enhances the kinase activity of TBK1 via its deacetylase activity

**Figure 4:**
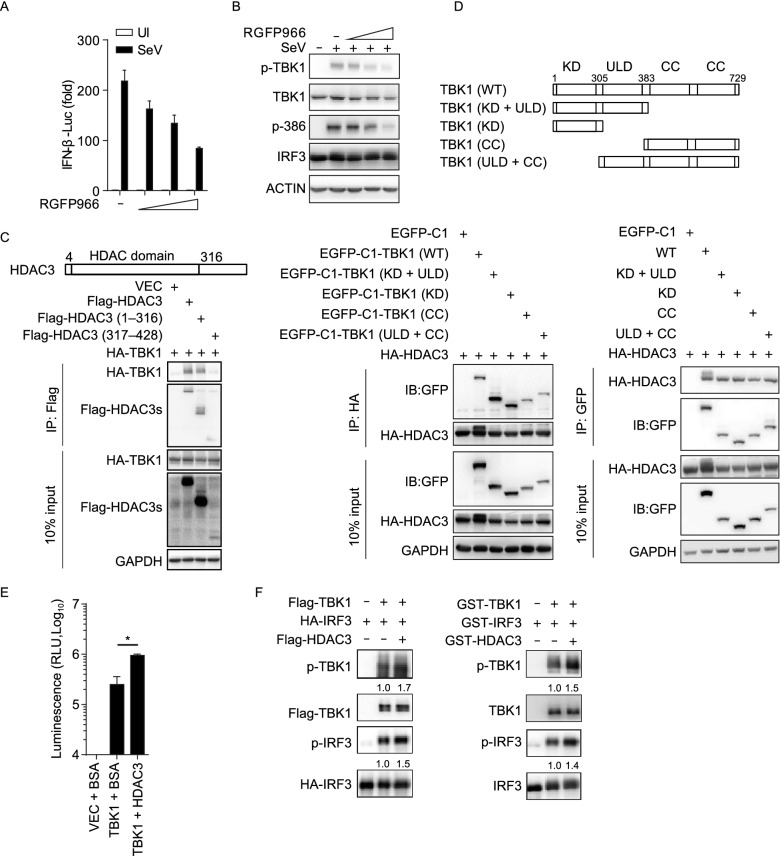
**HDAC3 directly enhances the TBK1 kinase activity via its deacetylase activity**. (A and B) Luciferase IFN-β reporter activity analyzing (A) and Immunoblot analysis of phosphorylated and total TBK1 or IRF3 (B) in HEK293T cells plus RGFP966 in dose (50, 100, 200 nmol/L), infected with SeV for 10 h. (C) Immunoblot analysis of HEK293T cells (1.5 × 10^6^) transiently co-transfected for 48 h with HA-tagged TBK1 together with vector encoding Flag-tagged wild-type HDAC3, HDAC3 (1–316 aa), or HDAC3 (317–428 aa) (above lanes), assessed before (10% input) or after (IP) immunoprecipitation with anti-Flag antibody. (D) Immunoblot analysis of HEK293T cells (1.5 × 10^6^) transiently co-transfected for 48 h with HA-tagged HDAC3 with vector encoding EGFP-tagged wild-type or mutant TBK1 (above lanes), assessed before (10% input) or after (Co-IP) Co-immunoprecipitation with the indicated antibodies. (E) TBK1 kinase activity assay combined with purified GST-HDAC3. HEK293T cells (4.0 × 10^5^) was transfected with vector or Flag-tagged TBK1. The expressed proteins immunoprecipitated by Flag tag antibodies and purified GST-HDAC3 or BSA were introduced into a mixture as indicated for TBK1 kinase activity assay. (F) *In vitro* kinase assay. HEK293T cells (4.0 × 10^5^) were transfected with Flag-tagged TBK1, HA-tagged IRF3 and Flag-tagged HDAC3 separately (left). The expressed proteins were immunoprecipitated by the indicated tag antibodies and incubated together as indicated. The GST-TBK1 purified protein and the peptide of IRF3 full length protein with kinase activity were introduced into a mixture containing recombinant purified HDAC3 (right). Data are representative of three independent experiments. Graphs show mean ± SD; *n* = 3. *P < 0.05; (Student’s *t*-test)

To analyze the mechanistic details, we first considered whether the deacetylation activity of HDAC3 played a critical role in inhibiting TBK1-mediated IRF3 activation. A reporter assay indicated that RGFP966, a specific inhibitor of HDAC3, could impair IFNB1 transcriptional activity and the phosphorylation of IRF3 at Ser386 and TBK1 at Ser172 in a dose-dependent manner during SeV infection (Fig. 4A and B). Furthermore, we mapped the domain of HDAC3 that interacted with TBK1. An HDAC3 mutant consisting of the HDAC domain alone was still capable of interacting with TBK1 (Fig. 4C). To determine how TBK1 interacted with HDAC3, we constructed TBK1 mutants with deletions in various domains and found that the kinase domain and coiled-coil domain of TBK1 were required for the interaction of TBK1 with HDAC3 (Fig. 4D).

To further investigate the effect of HDAC3 on the regulation of the kinase activity of TBK1, we performed an *in vitro* TBK1 kinase assay using a TBK1 kinase enzyme system, with or without HDAC3. As expected, HDAC3 markedly increased TBK1 kinase activity (Fig. 4E). We also introduced the fusion proteins containing HDAC3 (Flag-HDAC3) or recombinant HDAC3, Flag-tagged TBK1 and HA-tagged IRF3, or purified TBK1 and IRF3 protein into the *in vitro* kinase assay. Immunoblot (IB) analysis of IRF3 phosphorylation at Ser386 and TBK1 phosphorylation at Ser172 revealed that the phosphorylation of IRF3 or TBK1 was enhanced in the presence of HDAC3 (Fig. 4F). These results indicated that the deacetylase activity of HDAC3 could positively regulate TBK1 kinase activity.

### Lys241 and Lys692 deacetylation mediated by HDAC3 are critical for TBK1 kinase activity

**Figure 5:**
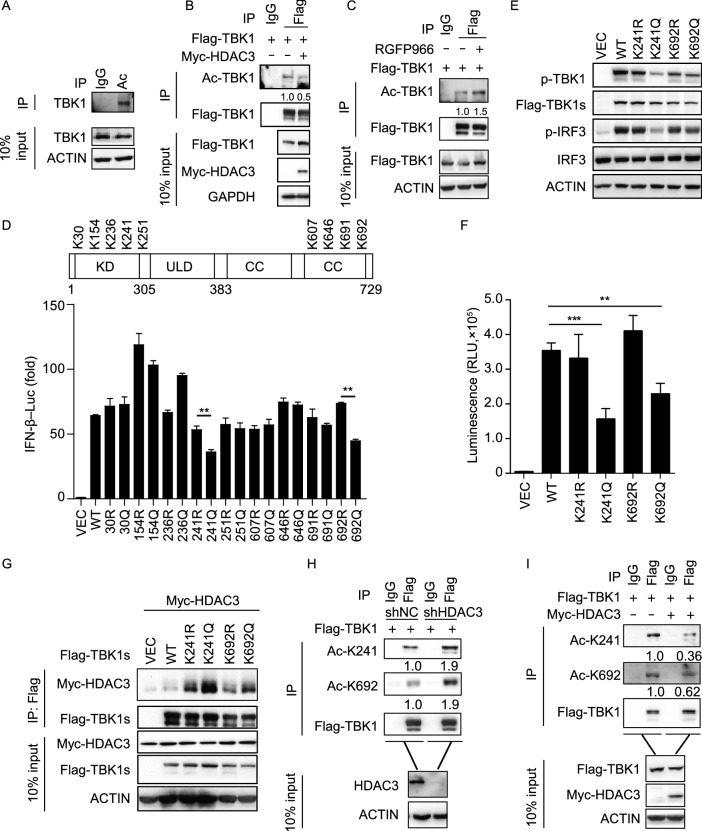
**Lys241 and Lys692 deacetylation mediated by HDAC3 are critical for TBK1 kinase activity**. (A) Immunoblot analysis of endogenous total TBK1 and acetylated TBK1 (Ac-TBK1) in HEK293T cells, assessed before (10% input) or after (IP) immunoprecipitation with IgG (control) or anti-acetyl-lysine. (B) Immunoblot analysis of acetylated TBK1 (Ac-TBK1) in HEK293T cells (1.5 × 10^6^) transiently co-transfected for 48 h with Flag-tagged TBK1 (3 μg) and empty vectors or Myc-tagged HDAC3 (1 μg) expression plasmids before co-immunoprecipitation (with anti-Flag or IgG as a control) and immunoblot analysis (with antibody to all acetylated lysine residues, anti-Flag, anti-Myc, anti-GAPDH). (C) Effect of HDAC3 specific inhibitor (RGFP966, 100 nmol/L) on TBK1 acetylation. Immunoblot analysis of acetylated TBK1 (Ac-TBK1) in HEK293T cells (1.5 × 10^6^), transiently transfected for 48 h with Flag-tagged TBK1 (3 μg) and treated for 24 h with RGFP966 before immunoprecipitation (with anti-Flag or IgG as a control) and immunoblot analysis (with antibody to all acetylated lysine residues, anti-Flag, anti-ACTIN). (D) Identification of lysine residues in TBK1 in HEK293T HDAC3-deficient cells, by immunoprecipitation combined with mass spectrometry, and Luciferase activity of an IFN-β reporter in HEK293T cells transfected with control vector (VEC) or wild-type TBK1 (WT) or mutants TBK1 (30R, 30Q, 154R, 154Q, 236R, 236Q, 241R, 241Q, 251R, 251Q, 607R, 607Q, 646R, 646Q, 691R, 691Q, 692R, 692Q). (E) Immunoblot analysis of phosphorylated and total TBK1, IRF3 in HEK293T cells transfected with control vector (VEC) or wild-type TBK1 (WT) or K241R K241Q, K692R, K692Q. (F) TBK1 kinase activity assay. HEK293T TBK1-knockout cells (4 × 10^5^) transiently transfected for 48 h with Flag-tagged empty vector (VEC), wild-type TBK1 (WT) or TBK1 mutants (K241R, K241Q, K692R or K692Q) before immunoprecipitation (with anti-Flag), then according the TBK1 kinase activity kit manufacture. (G) Interaction between HDAC3 and wild type (WT) or TBK1 mutants. Immunoblot analysis of HEK293T cells (1.5 × 10^6^) transiently co-transfected for 48 h with Myc-tagged HDAC3 and Flag-tagged empty vector, wild type (WT) or TBK1 mutants (K241R, K241Q, K692R, K692Q) (3 μg each) before co-immunoprecipitation (with anti-Flag) and immunoblot analysis were performed with the indicated antibodies. (H and I) Immunoblot analysis of specific sites of acetylated TBK1 (Ac-K241, Ac-K692) in HDAC3 knockdown HEK293T cells (1.5 × 10^6^) (H) or HDAC3 overexpressed HEK293T cells (1.5 × 10^6^) (I) transiently transfected for 48 h with Flag-tagged TBK1 (3 μg each) and empty vectors or Myc-tagged HDAC3 (1 μg in I) expression plasmids before immunoprecipitation (with anti-Flag or IgG as a control) and immunoblot analysis (with antibody to TBK1 acetylated at Lys241 (K241Ac) or K692Ac, anti-Flag, anti-HDAC3 or anti-Myc). Data are representative of three independent experiments. Graphs show mean ± SD; *n* = 3. **P < 0.01; ***P < 0.001 (Student’s *t*-test)

We assessed the lysine acetylation of endogenous TBK1 immunoprecipitated from HEK293T cells in the absence of stimuli and found that endogenous TBK1 was distinctly acetylated (Fig. 5A). The acetylation of TBK1 was reduced dramatically following HDAC3 overexpression (Fig. 5B). Correspondingly, TBK1 acetylation gradually increased when the cells were treated with the HDAC3 specific inhibitor RGFP966 (Fig. 5C). To identify definitively acetylated lysine residues in TBK1, we used an antibody against TBK1 to purify endogenous TBK1 from whole cell extracts of HDAC3-deficient HEK293T cells infected with SeV. According to the reports and mass spectrometry analysis revealed that nine lysine residues of TBK1 were modified by acetylation: Lys30, Lys154, Lys236, Lys241, Lys251, Lys607, Lys646, Lys691, and Lys692 (Fig. 5D), all of which were distributed either in the kinase domain or the coiled-coil domain of TBK1 (Li et al., 2016). Both of these domains were the key domains through which TBK1 interacted with HDAC3. We then sought to determine whether the acetylation sites of TBK1 noted above were able to regulate the kinase activity of TBK1. Each of the identified lysine residues was mutated into a glutamine (K to Q) or arginine (K to R) (to mimic the acetylated or non-acetylated state of the lysine residue), followed by transient transfection and reporter assays. Compared to other mutations, the K241Q and K692Q mutants had an impaired ability to activate the IFN-β reporter, whereas the K241R and K692R mutants still retained partial ability to activate the IFN-β luciferase reporter (Fig. 5D). In parallel experiments, the K241Q or K692Q mutants showed reduced phosphorylation of IRF3, but the K241R or K692R mutants had a limited impact compared to the wild type (Fig. 5E). An *in vitro* TBK1 kinase assay using a TBK1 Kinase Enzyme System showed a higher luciferase value, indicating increased kinase activity in K241R and K692R mutants as compared to the K241Q and K692Q mutants (Fig. 5F). We then found that the K241Q or K692Q mutants had stronger interactions with HDAC3 than the K241R or K692R mutants (Fig. 5G). To further confirm the role of acetylation at Lys241 and Lys692, we generated specific antibodies against TBK1 acetylated at Lys241 or Lys692. Dot-blot analysis or Western blot indicated that the antibodies specifically recognized TBK1 peptide acetylated at Lys241 or Lys692, but did not recognize the control peptide or the K241R or K692R mutants (Fig. S4A–C). After transfecting the Flag-tagged TBK1, we found Lys241 or Lys692 acetylated TBK1, and the acetylation of TBK1 at Lys241 or Lys692 was increased in HDAC3-knockdown HEK293T cells and reduced in HDAC3 overexpressed HEK293T cells (Fig. 5H and 5I). Thus, HDAC3 mediated the deacetylation of TBK1 at Lys241 and Lys692 to promote TBK1 kinase activity.

### Lys241 is critical for the interaction between TBK1 and IRF3

**Figure 6:**
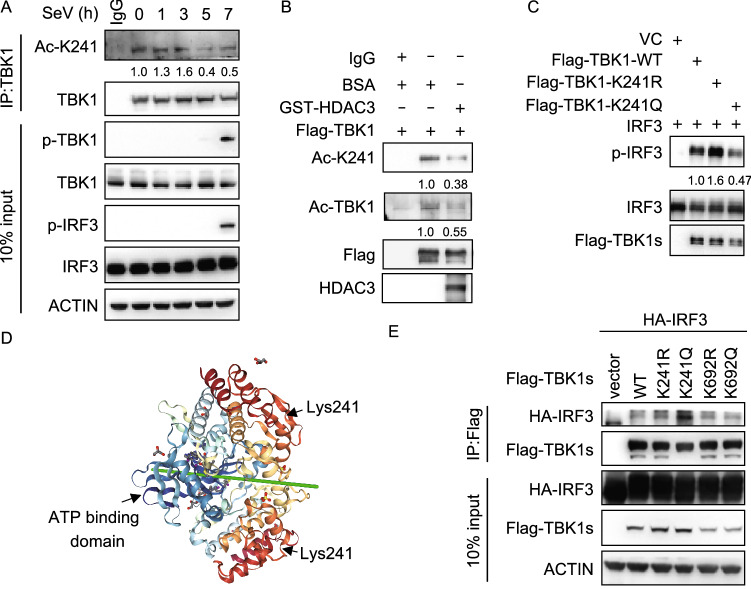
**Lys241 is critical for the interaction between TBK1 and IRF3**. (A) Immunoblot analysis of endogenous acetylation of TBK1 at Lys241 (Ac-K241) in HEK293T cells, followed by infection with SeV for 0–7 h, before co-immunoprecipitation (with anti-TBK1 or IgG as a control) and immunoblot analysis with anti-Ac-K241, anti-phosphorylated and total TBK1 or IRF3. (B) *In vitro* deacetylation assay. The acetyl-modified TBK1 immunoprecipitated and purified recombinant HDAC3 were performed as indicated for deacetylation assay *in vitro*. Immunoblot analysis with antibody to TBK1 acetylated at Lys241 (Ac-K241), anti-total acetylation, anti-Flag, anti-HDAC3. (C) Effect of TBK1 K241 mutant on kinase assay *in vitro.* HEK293T cells (4 × 10^5^) were transfected with Flag-tagged empty vector (VEC), wild type (WT) or Flag-TBK1-K241R, or Flag-TBK1-K241Q separately. The expressed proteins were immunoprecipitated by the Flag tag antibodies and incubated with recombinant IRF3 protein together as indicated. (D) Structure of TBK1 kinase domain. (E) The interaction between TBK1 K241, K692 mutants and IRF3. Immunoblot analysis of HEK293T cells (1.5 × 10^6^) transiently co-transfected for 48 h with HA-IRF3 and Flag-tagged empty vector (VEC), wild type (WT) or K241R, K241Q, K692R, K692Q before co-immunoprecipitation (with anti-Flag) and immunoblot analysis were performed with the indicated antibodies. Data are representative of three independent experiments

By using a specific antibody against acetylated lysine residues, we analyzed Lys241-acetylated TBK1 immunoprecipitated from HEK293T cells during SeV infection. We detected that TBK1 was acetylated at Lys241 in HEK293T cells. We also found that the acetylation of TBK1 at Lys241 was strengthened and then weakened, and the level of acetylation was highest 3 hours post-infection with SeV (Fig. 6A). In an *in vitro* deacetylation assay, HDAC3 dramatically deacetylated TBK1 at Lys241 as compared to the control (Fig. 6B). In an *in vitro* kinase assay, we found that the K241R TBK1 mutant was better at mediating the *in vitro* phosphorylation of recombinant IRF3 (Fig. 6C). Thus, deacetylation of Lys241 was critical for the kinase activity of TBK1. Lys241 is located at the outer of the “pocket” of the kinase domain (Fig. 6D). We surmised that Lys241 could affect substrate binding, such as IRF3. To test this hypothesis, we analyzed the interaction between K241R or K241Q with IRF3. As shown in Fig. 6E, K241Q had a stronger interaction with IRF3 as compared to K241R. Together, these results suggested that the deacetylation of Lys241 in TBK1 was important for its interaction with IRF3 and its kinase activity.

### Lys692 deacetylation controls the dimerization of TBK1

**Figure 7:**
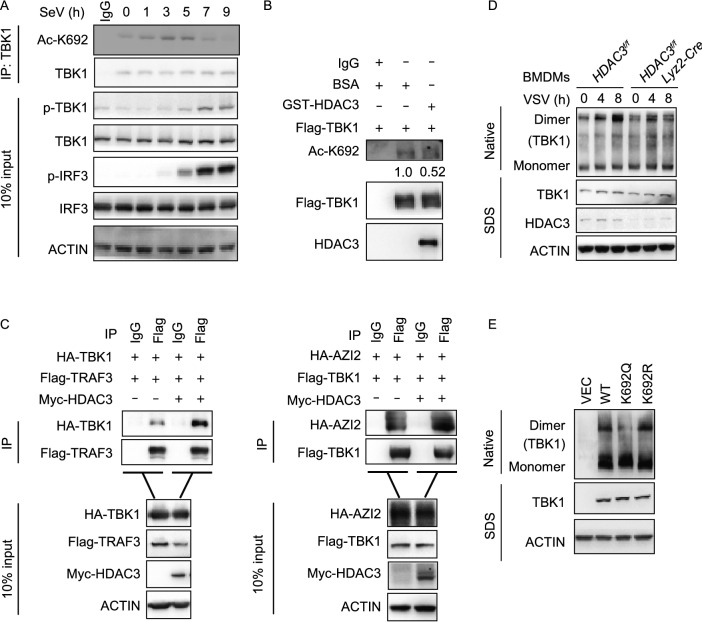
**Lys692 deacetylation controls the dimerization of TBK1**. (A) Immunoblot analysis of endogenous acetylation of TBK1 at Lys692 (Ac-K692) in HEK293T cells (1.5 × 10^7^), followed by infection with SeV for 0–9 h, before co-immunoprecipitation (with anti-TBK1 or IgG as a control) and immunoblot analysis with anti-Ac-K692, anti-phosphorylated and total TBK1or IRF3. (B) *In vitro* deacetylation assay. Assays were performed as in Fig. 6B. Immunoblot analysis with antibody to TBK1 acetylated at Lys692 (Ac-K692), anti-Flag, anti-HDAC3. (C) Effect of HDAC3 overexpression on TRAF3-TBK1, AZI2-TBK1 association. Immunoblot analysis of HEK293T cells (1.5 × 10^6^) transiently co-transfected for 48 h with the indicated plasmids before co-immunoprecipitation (with anti-Flag or IgG as a control) and immunoblot analysis were performed with the indicated antibodies. (D) Immunoblot analysis of TBK1 in dimerization in *HDAC3*^*fl*/*fl*^ or *HDAC3*^*fl*/*fl*^ *Lyz2-Cre* BMDMs and left infected with VSV for the indicated times, followed by native PAGE. (E) Immunoblot analysis of TBK1 in dimer or monomer form in HEK293T TBK1-knockout cells (4 × 10^5^), transiently transfected for 48 h with Flag-tagged empty vector (VEC), wild type (WT) or TBK1 mutants (K692Q or K692R), followed by native PAGE. Data are representative of three independent experiments

We also analyzed acetylation of TBK1 at Lys692 during SeV infection. We found that the acetylation of TBK1 at Lys692 was strengthened and then weakened similar to Lys241, and the level of acetylation was highest at 3–5 hours post-infection with SeV (Fig. 7A). In an *in vitro* deacetylation assay, HDAC3 dramatically deacetylated TBK1 at Lys692 as compared to the control (Fig. 7B). Lys692 is located in the coiled-coil domain of TBK1, which is exclusively responsible for the association with the adaptor proteins. Not surprisingly, HDAC3 promoted the interaction between TBK1 and TRAF3 or AZI2, the adaptor proteins of TBK1 (Fig. 7C). Indeed, knockout of HDAC3 had the opposing effects (Fig. S5A and S5B). However, K692R or K692Q showed no different interaction with IRF3 as compared to the wild-type (Fig. 6E). We found that the dimerization of TBK1 was lower in *HDAC3*^*fl*/*fl*^ *Lyz2-Cre* peritoneal macrophage after infection with VSV (Fig. 7D). The dimerization was enhanced in K692R, but was reduced in K692Q as compared to the wild-type (Fig. 7E). To investigate the role of Lys692 deacetylation in dimerization, we constructed the mutants TBK1-355A/357A/547D to prevent dimerization of TBK1 (Larabi et al., 2013). When the dimerization was abolished, the acetylation of Lys692 was increased, but not the acetylation of Lys241 (Fig. S6A and S6B). Together, these results indicated that the deacetylation of Lys692 mediated by HDAC3 decided the dimerization of TBK1.

### TBK1 phosphorylates HDAC3 to promote its deacetylase activity

**Figure 8:**
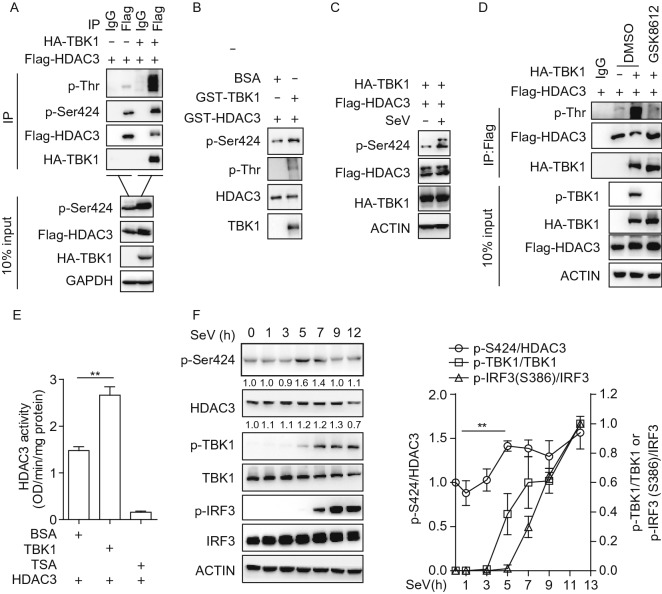
**TBK1 phosphorylates HDAC3 to promote its deacetylase activity**. (A) Immunoblot analysis of phosphorylated HDAC3 by TBK1 in HEK293T cells (1.5 × 10^6^) transiently co-transfected for 48 h with Flag-tagged HDAC3 and empty vectors or HA-tagged TBK1 expression plasmids before co-immunoprecipitation (with anti-Flag or IgG as a control) and immunoblot analysis (with antibody to anti-Phospho-HDAC3 (Ser424), anti-Phospho-Threonine, anti-HA or anti-Flag). (B) *In vitro* kinase assay of phosphorylated HDAC3. The peptide of HDAC3 full length protein as the substrate and combinations of BSA or recombinant protein TBK1 were introduced as indicated and the immunoblot analysis with antibody to anti-Phospho-HDAC3 (Ser424), anti-Phospho-Threonine, anti-HDAC3 or anti-TBK1. (C) Immunoblot analysis of HEK293T cells (2 × 10^5^) transiently co-transfected for 48 h with HA-tagged TBK1 (1 μg) and Flag-tagged HDAC3 (300 ng), followed by infection with or without SeV for 6 h. (D) HDAC3 was phosphorylated by TBK1. HEK293T cells (1.5 × 10^6^) were transiently co-transfected for 48 h with Flag-tagged HDAC3 and empty vectors or HA-tagged TBK1 expression plasmids, and treated with GSK8612 for 1 h before co-immunoprecipitation (with anti-Flag or IgG as a control) and the immunoblot analysis with antibody to anti-Phospho-Threonine, anti-HA, anti-Flag or anti-phospho-TBK1. (E) Analysis of HDAC3 enzymatic activity in the presence of recombinant TBK1 protein or TSA (negative control). (F) Immunoblot analysis of phosphorylated and total HDAC3, TBK1 or IRF3 and β-actin of HEK293T cells infected with SeV for 0–12 h (left) and densitometric analysis (right). Data are representative of three independent experiments. Graphs show mean ± SD; *n* = 3. **P < 0.01 (Student’s *t*-test)

HDAC3 activity is regulated by phosphorylation at Ser424 (Zhang et al., 2005). We suspected that HDAC3 might be phosphorylated as a substrate of TBK1. To test this hypothesis, we used the fusion proteins containing HDAC3 and TBK1 in an *in vitro* kinase assay. IB analysis of phospho-serine/threonine or the phospho-HDAC3 at Ser424 showed that HDAC3 could be phosphorylated by TBK1 directly (Fig. 8A and 8B). The phosphorylation at Ser424 was enhanced during SeV infection (Fig. 8C). When TBK1 was treated with its inhibitor GSK8612, the phosphorylation of HDAC3 was significantly decreased (Fig. 8D). To determine whether TBK1 influenced the enzymatic activity of HDAC3, we performed an *in vitro* HDAC3 deacetylation assay using an HDAC activity direct assay kit. As expected, TBK1 markedly increased HDAC3 deacetylation activity (Fig. 8E). Then we analyzed the phosphorylation of HDAC3 in HEK293T cells infected with SeV. The phosphorylation at Ser424 was significantly increased, which was consistent with TBK1 activation. We then analyzed the ratio of HDAC3, TBK1 or IRF3 phosphorylation relative to controls. As shown in Fig. 8F, there was transient increase in HDAC3 phosphorylation at 5–7 h post SeV infection, which preceded the phosphorylation of TBK1 or IRF3. These results indicated the TBK1 could phosphorylate HDAC3 to promote its deacetylase activity.

## Discussion

The TBK1-IRF3 axis is critical for the innate immune response. We have shown here that HDAC3 plays an essential role in the innate immune response *in vitro* and *in vivo*. HDAC3 deacetylated TBK1 to promote the dimerization and activation of TBK1. However, the acetylation of TBK1 at Lys241 during the early stage of viral infection enhanced the recruitment of IRF3 to TBK1. TBK1 phosphorylated HDAC3 to enhance HDAC3 deacetylation activity and TBK1 activation. Thus, dynamic regulation of TBK1 acetylation and deacetylation plays a key role in the activation of TBK1 and the production of type I IFNs. Our study provides a novel insight into how acetylation tightly regulates TBK1 activation and antiviral responses.

It was reported that HDACs can act as both positive and negative regulators in TLRs- or virus-triggered innate immune responses (Aung et al., 2006; Nusinzon and Horvath, 2006; Halili et al., 2010). HDAC1 and HDAC8 repress the transcription of *Ifnb1* mRNA by attenuating the acetylation of histone H3/H4 in *Ifnb1* promoter, while HDAC6 mediates the deacetylation of RIG-I for viral RNA detection and the deacetylation of β-catenin for IRF3-activated transcription (Liu et al., 2016). Independent of its deacetylase activity, HDAC4 blocks the phosphorylation of IRF3 to repress IRF3-mediated IFN-beta expression (Yang et al., 2018). Here, we provided evidence that HDAC3 positively regulates innate immunity as a deficiency in HDAC3 repressed the production of type I IFNs and antiviral responses both *in vitro* and *in vivo*. HDAC3 directly deacetylated TBK1 at Lys241 and Lys692 and activated TBK1 to promote IRF3-activated transcription. HDAC3 is known to shuttle between the nucleus and cytoplasm (Yang et al., 2002; Park et al., 2014). In the nucleus, HDAC3 deacetylates histones and regulates the transcription of different genes (Zhang et al., 2005; Karagianni and Wong, 2009). The HADC3 mutant (HDAC3-NLS), which resides in the nucleus, did not affect the phosphorylation of TBK1 and IRF3, indicating that HDAC3 does not regulate TBK1 activation through the epigenetic modification of chromatin. It has been reported that HDAC9 is involved in the deacetylation of TBK1 (Li et al., 2016). HDAC9, a class IIa HDAC, possesses limited deacetylase activity and exhibits activity only when associated with a catalytically active class I HDAC family member, such as HDAC3 (Fischle et al., 2002; Lahm et al., 2007; Chen et al., 2015). Thus, HDAC3 is a key molecule that regulates TBK1 deacetylation to enhance its kinase activity. HDAC3 also positively regulates the IFN-I-mediated signaling pathway (Feng et al., 2018), which further highlights the functional importance of HDAC3 in the innate immune response and antiviral responses.

As a critical kinase involved in innate immunity, the activity of TBK1 is tightly regulated to maintain immune homeostasis. K63-linked polyubiquitination of TBK1 at Lys30 and Lys401 and autophosphorylation of TBK1 at Ser172 were shown to be critical for its activation (Wang et al., 2009; Li et al., 2011; Ma et al., 2012; Tu et al., 2013; Shevtsova et al., 2016). Modification of TBK1 at Lys694 by the small ubiquitin-like modifier SUMO contributes to the ability of TBK1 to bind to adaptors for the transduction of IFN signaling (Saul et al., 2015). Here, we found that both acetylation and deacetylation were critical for the activation of IRF3 by TBK1, independent of other reported conventional PTMs. During the resting stage, acetylation at Lys241 or Lys692 was able to be detected, but double acetylation of Lys241 and Lys692 was minimal. After induction with stimuli, TBK1 was acetylated at Lys241 and Lys692, and the acetylation level was enhanced at the early stage. We found that the acetylation at Lys241 or Lys692 was independent of one another, as K241R or K692R, mimics of deacetylation, had little effect on the acetylation of the other (Fig. S7A). During induction with virus, the double acetylation of Lys241 and Lys692 was accumulated (Fig. S7B). K241Q, a mimic of acetylation, showed a stronger interaction with IRF3 as compared to the wild-type or K241R, implying that the acetylation of TBK1 at Lys241 enhanced the recruitment of IRF3. However, K241Q had limited kinase activity, which was consistent with the fact that knockdown of HDAC3 repressed IFN production. Thus, the acetylation of TBK1 at Lys241 is an early step before the activation of TBK1. The precise quantitative analysis of TBK1 K241 acetylation is limited by existing technology. It’s very difficult to provide the specific data about how much TBK1 K241 was acetylated at the beginning of time course. We only found the acetylation of K241 in uninfected cell was lower compared with Lys241 acetylation at 1–3 h post SeV infection (Fig. 6A). Further research is needed to identify which acetylase(s) is involved in the modification and the function of other acetylation sites.

HDAC3-mediated deacetylation of TBK1 at Lys241 and Lys692 occurred during the late stage after SeV challenge (5 hours post-infection). The K241R TBK1 mutant demonstrated a stronger ability to phosphorylate IRF3 *in vitro* as compared with WT TBK1. Thus, deacetylation of TBK1 at Lys241 enhanced the activation of TBK1. A K692R mutant was able to enhance the dimerization of TBK1 as compared with the K692Q mutant. Thus, HDAC3 mediated the deacetylation of Lys692, which contributed to the dimerization and kinase activity of TBK1. Moreover, the acetylation and deacetylation at Lys241 and Lys692 were coordinated and dynamically regulated, which is essential for the innate immune response.

The phosphorylation of HDAC3 at Ser424 can significantly enhance the deacetylase activity of HDAC3 (Zhang et al., 2005). We found that HDAC3 could be phosphorylated by TBK1. The phosphorylation level of HDAC3 at Ser424 increased at a time point that was consistent with the activation of TBK1. HDAC3 was able to also positively regulate the IFN-STAT pathway (Klampfer et al., 2004). The feedback of activation of HDAC3 by TBK1 was able to further enhance IFN production and IFN-STAT activation.

Altogether, we demonstrated not only a new mechanism for the activation of TBK1 and the innate immune response but also that HDAC3 is a precise regulator of the cellular antiviral response.

## Methods

### Reagents and antibodies

Poly I:C (tlrl-pic), 2’3’-cGAMP (tlrl-nacga23-1), CpG ODN (tlrl-2006-1) were from InvivoGen (France). Lipopolysaccharide (LPS, S1732) was from Beyotime Biotechnology (China). Protein G-agarose (#16-266) used for immunoprecipitation was from Millipore (USA). HDAC3 inhibitor RGFP966 (S7229) was from Selleck (USA). TBK1 inhibitor GSK8612 (#2361659-62) was from MCE (China). The anti-bodies used in this study are in Table S1. The antibody directed against TBK1 acetylated at Lys241 (TBK1 K241Ac) and Lys692 (TBK1 K692Ac) were customized produced by Abmart (Shanghai, China). Antigenic 11 aa peptide (amino acids 234–244 of TBK1) with acetylated lysine (CYKIIGK(Ac)PSG) and the according control peptide (CYKIIGKPSG) were designed and *in vitro* synthesized. Antigenic 11 aa peptide (amino acids 685–695 of TBK1) with acetylated lysine (CMTLGMKK(Ac)LKE) and the according control peptide (CMTLGMKKLKE) were designed and *in vitro* synthesized. The rabbit polyclonal antibodies to the peptides were purified using protein A.

### Mice

All animal procedures in this investigation conform to the Guide for the Care and Use of Laboratory Animals published by the National Institutes of Health (NIH publication 85-23, revised in 1996) and the approved regulations were set by the Laboratory Animal Care Committee at Wuhan Institute of Virology, Chinese Academy of Sciences (CAS) (permit WIVA02201703). *Lyz2-Cre* mice on the C57BL/6 background were from Model Animal Research Center of Najing University. *HDAC3*^*fl*/*fl*^ mice on the C57BL/6 background were provided by Prof. Yujun Shi (Sichuan University). All of the mice were bred and maintained under specific-pathogen free animal facility in Wuhan Institute of Virology, CAS.

### Cells and virus

All cell cultures were maintained in a humidified atmosphere at 37 °C with 5% CO_2_. The HEK293T, Raw264.7, THP-1 and Vero cell were from American Type Culture Collection (ATCC), and cultured in Dulbecco’s modified Eagle medium (DMEM) (Invitrogen, USA) supplemented with 2 mmol/L L-glutamine, nonessential amino acids, 10% fetal bovine serum (FBS) (Invitrogen), and 1% penicillin-streptomycin (Life Technologies, USA). THP-1 cells were cultured in RPMI 1640 (Hyclone, USA) supplemented with 10% fetal bovine serum (FBS) (Invitrogen), and 1% penicillin-streptomycin (Life Technologies, USA).

Sendai virus (SeV) and vesicular stomatitis virus VSV-GFP were provided by Prof. Hanzhong Wang, HSV-1 with GFP was provided by Prof. Chunfu Zheng. All virus were amplified and titrated were as previously described. Cells were infected with SeV (1 M.O.I.), VSV (1 M.O.I) or HSV-1 (10 M.O.I.) for the indicated time. Cells were stimulated with poly(I:C) (50 μg/mL), LPS (100 ng/mL), cGAMP (1 μg/mL), CPG ODN (1 μg/mL) for the indicated time.

### CRISPR-Cas9 knockout

Double-stranded oligonucleotides corresponding to the target sequences were cloned into the lenti-CRISPR-V2 vector and co-transfected packaging plasmids into HEK293 cells. 48 h after transfection, the viruses were harvested, ultra-filtrated (0.22 mm filter, Millipore) and used to infect HEK293T or THP1 cells in the presence of polybrene (8 μg/mL). The infected cells were selected with puromycin (2 μg/mL) for at least 5 days. Human HDAC3 and TBK1 sgRNA targeting sequences were in Table S4.

### Isolation of macrophages

BMDMs were isolated from the mouse tibia and femur of wild-type and HDAC3 deficient mice and cultured in 10-cm Petri dish at 37 °C for 6 days. 10 mL RPMI medium 1640 (supplemented with 10% FBS, L-glutamine, and 30% L929 supernatant) was added at day 4.

### Plasmid constructs and transfection

Mammalian expression plasmids for HA-, Flag-, Myc-tagged HDAC3 and its Flag-tagged deletion mutants were constructed by standard molecular biology techniques. Mammalian expression plasmids for HA- or Flag-tagged TBK1 and all Flag-tagged site-directed mutagenesis of TBK1 were amplified and inserted into the pXJ40 vectors. All constructs were confirmed by sequencing and a complete list of primers was provided in the supplementary materials (Table S2). Mammalian expression plasmids for RIG-I, VISA, IRF3-5D were provided by Prof. Hongbing Shu. IFN-β, Interferon-stimulated response element (ISRE) promoter luciferase reporter plasmids were purchased from Clontech (USA). pRL-TK was purchased from Promega (USA). The plasmids for EGFP-C1-TBK1 deletion mutants, HA-tagged TANK, HA-tagged AZI2 were constructed by our lab. Plasmids were transiently transfected into HEK293T cells with using Lipofectamine 2000 reagents (Invitrogen, USA) following the manufacturer instructions.

### Luciferase assays

HEK293T (1 × 10^5^) in 24-well plates were transfected with IFN-β or ISRE-firefly luciferase reporter (100 ng) and Renilla luciferase reporter (10 ng) together with HDAC3 or empty control plasmid in dose. 24 h later, the cells were stimulated with virus for 10 h, and luciferase activities were measured with Dual-Glo® Luciferase Assay System (E1960, Promega, USA). Data were normalized by calculating the ratio between firefly luciferase activity and Renilla luciferase activity.

### ELISA

Secreted cytokines in cell culture supernatants or sera from virus infected-mice were analyzed using human IFN-β (CUSABIO, China), mouse IFN-β (Biolegend, China) ELISA kits according to the manufacturer’s instructions.

### RNA-mediated interference and lentiviral transduction

HEK293T (4 × 10^5^) in 6-well plates were transfected with siRNA (20 nmol/L) by using Lipofectamine RNAiMAX reagent (Invitrogen, USA). The following sequences targeting for HDAC3 were designed and synthesized by Qiagen (Germany). The lentiviruses were produced in 293T cells by co-transfection of the shNC or shHDAC3 and packaging vectors psPAX2 and pMD2.G (Bought from addgene), (shRNAs were cloned into the lentivirus vector pLKO.1 plasmid). 48 h or 72 h post-transfection, the supernatant was collected and applied to infect target cells, followed by cell selection through puromycin (1 μg/mL). The information of the sequences was in Table S4.

### RNA quantification

Total RNA was extracted with Trizol reagent following the manufacturer’s protocols (Invitrogen, USA). Specific mRNAs were quantified by one-step real-time RT-PCR using the QuantiFast SYBR Green RT-PCR kit (Qiagen, Germany). The data were normalized to the expression of the β-actin for each individual sample. The 2^−ΔΔCt^ method was used to calculate relative expression changes. The primer sequences for quantitative RT-PCR are provided in Table S3.

### Co-immunoprecipitation and immunoblot analysis

For co-immunoprecipitation and immunoblot analysis, cells were lysed with lysis buffer (50 mmol/L Tris, pH 7.5, 1 mmol/L EGTA, 1 mmol/L EDTA, 1% Triton X-100, 150 mmol/L NaCl, 100 μmol/L phenylmethylsulfonyl fluoride (PMSF), and a protease inhibitor cocktail (Complete Mini, Roche, Swiss) for 30 min in 4 °C. Cell lysates were centrifuged at 13,000 ×*g* for 10 min at 4 °C and quantified using the Bradford method (#500-0006, Bio-Rad, USA). For Western blot, the supernatants were recovered and boiled in 5× loading buffer. For immunoprecipitation, the supernatants were collected and incubated with protein G agarose beads recovered and mixed with specific antibodies for overnight with rotation at 4 °C. Protein G agarose-bound immune complexes were collected by centrifugation at 5,000 ×*g* for 1 min, washed at least five times with IP buffer, and boiled in 2× loading buffer. Samples were separated by SDS-PAGE, electro-transferred to nitrocellulose filter membranes (Millipore, USA) and then blocked for 1 h with 5% nonfat milk solution, followed by blotting with the primary antibodies. The proteins were visualized using suitable HRP-conjugated secondary antibodies (Jackson Immuno Research, USA) and SuperSignal-Femto chemiluminescent substrate (Pierce, USA).

### *In vitro* kinase assay

HEK293T (1.5 × 10^6^) cells were transfected with indicated plasmid separately. The expressed proteins were immunoprecipitated by the indicated tag antibodies. The immune-complexes were washed twice with 1× cell lysis buffer (#9803s, CST, USA) and then twice with 1× kinase buffer (#9802, CST, USA). Kinase reactions were performed by incubation of immune-complexes or purified proteins as indicated with 1× kinase buffer, 10 mmol/L ATP (#9804, CST, USA) and at 30 °C for 90 min in 50 μL reaction mixture. Samples were separated by SDS-PAGE, and analyzed by immunoblotting with anti-phospho-IRF3 and anti-phospho-TBK1.

### *In vitro* deacetylation assay

HEK293T (4 × 10^5^) cells in 6-well plates were transfected with Flag-tagged vector, or WT, or TBK1 mutants (241R, 241Q, 692R, 692Q). The expressed proteins were immunoprecipitated by Flag antibody. The immunoprecipitate were washed with deacetylation buffer (50 mmol/L Tris-HCl (pH 8.0), 50 mmol/L NaCl, 10% glycerol, 0.5 mmol/L EDTA, 1 mmol/L dithiothreitol, 10 mmol/L sodium buytrate and 1 mmol/L phenylmethylsulfonyl fluoride). Deacetylation reactions were performed by incubation of immunoprecipitate and purified GST-tagged HDAC3 protein as indicated with 1× deacetylation buffer, at 37 °C for 90 min in 50 μL reaction mixture. Samples were separated by SDS-PAGE, and analyzed by immunoblotting with K241Ac, K692Ac antibodies.

### Mass spectrometry

HEK293T (1 × 10^7^) cells were infected with SeV and then lysed for immunoprecipitation with antibody to acetyl (or with IgG as a control). After Commassie Blue staining, the TBK1 specific band with intensive signal compared with IgG control were cut and then used as the sample for mass spectrometry.

### TBK1 kinase activity assay

HEK293T TBK1 KO (4 × 10^5^) cells in 6-well plates were transfected with Flag-tagged vector, or WT, or TBK1 mutants (241R, 241Q, 692R, 692Q). 48 h after transfection, the cells were lysed for immunoprecipitation with antibody to Flag. The TBK1 kinase activity was analyzed using TBK1 Kinase Enzyme System (V8291, Promega, USA). TBK1 kinase activity assay according to the manufacturer’s instructions.

### IRF3 and TBK1 dimer assay

Native PAGE was performed with an 8% acrylamide gel without SDS. The gel was pre-run for 60 min at 200 V on ice with 25 mmol/L Tris-HCl (pH 8.4) and 192 mmol/L glycine with or without 0.5% deoxycholate in the cathode buffer and anode buffer, respectively. Samples in the 5× loading buffer (50 mmol/L Tris-HCl, pH 6.8, 0.002% Bromphenol Blue, and 15% glycerol) were applied on the gel and underwent electrophoresis for 60 min at 200 V on ice, followed by immunoblot analysis.

### Statistical analysis

Differences between groups were evaluated using the two-tailed, unpaired Student’s *t*-test available in the GraphPad Prism 5 software package (GraphPad Software, Inc.). Coprecipitation efficiency and fluorescence images were analyzed using ImageJ (NIH). *P*-values were calculated, and statistical significance was reported as highly significant with *P < 0.05). Analytic results are presented as mean ± SD.

## Electronic supplementary material

Below is the link to the electronic supplementary material.

Supplementary material 1 (PDF 1034 kb)
