# Supplementary material for: Histone deacetylase 3 promotes innate antiviral immunity through deacetylation of TBK1
Source: Protein Cell. 2020 Aug 9;12(4):261–78. doi: 10.1007/s13238-020-00751-5 (PMC8018997; doi:10.1007/s13238-020-00751-5)
Supplement: Supplementary file 1 — Supplementary material 1 (PDF 1034 kb) [file 13238_2020_751_MOESM1_ESM.pdf]

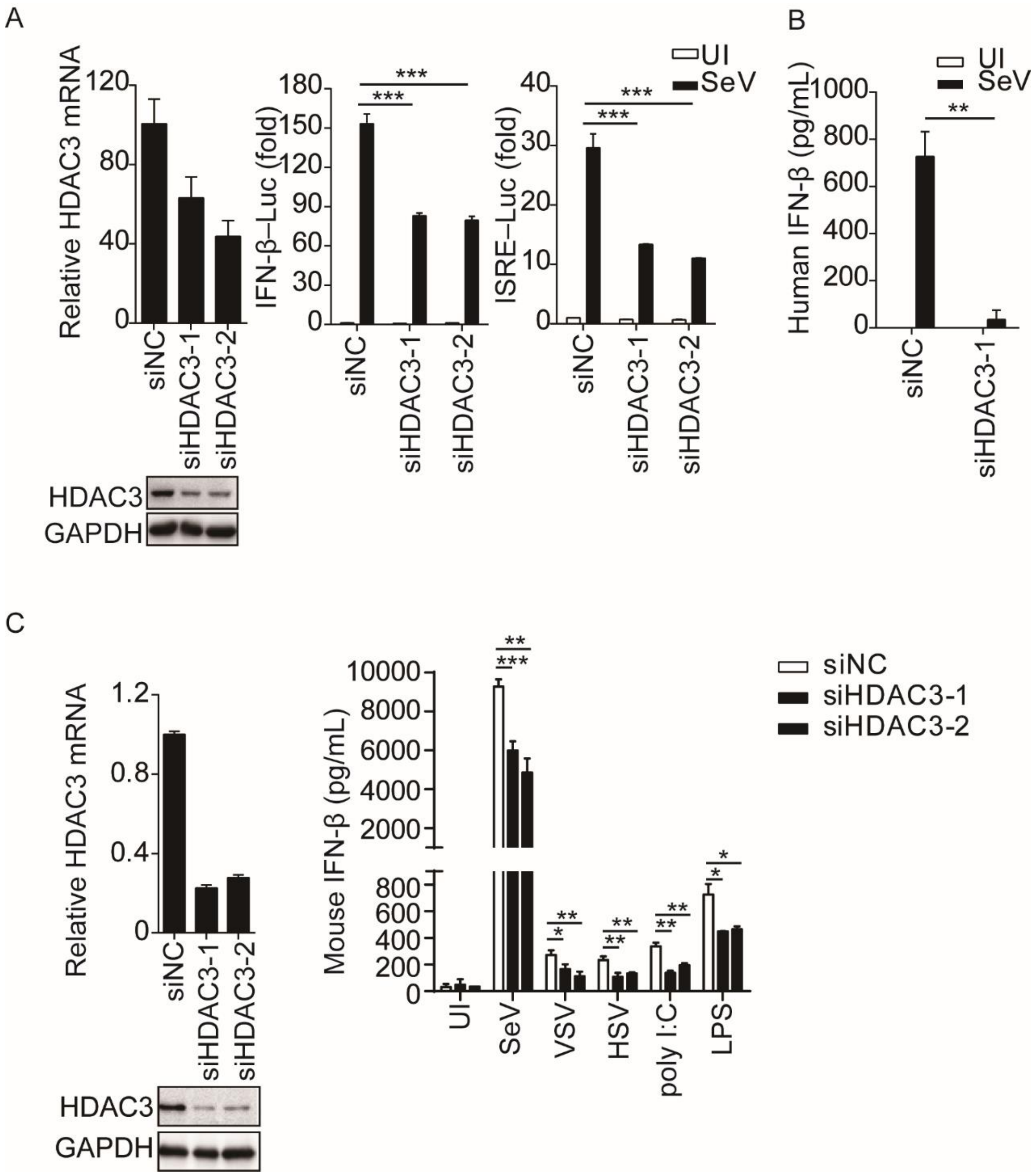

Supplementary Figure 1. Silencing of HDAC3 impairs the production of Type I Interferon. (A) Luciferase activity in HEK293T cells (1×10^5), co-transfected for 36 h with ctrl siNC or siHDAC3 (siHDAC3-1, siHDAC3-2) and a luciferase reporter for IFN-β (middle) (IFN-β-luc) or for ISRE (right) (ISRE-luc) , uninfected or infected with SeV for 10 h before luciferase assays were performed. HDAC3-Knockdown efficiency in the HEK293T cells were confirmed by Quantitative RT-PCR analysis

(left) and immunoblotting analysis (bottom). **(B)** ELISA analysis of IFN-β in the supernatant of HEK293T cells (2×10^5) transfected for 36 h with ctrl siNC or siHDAC3-1, followed by infection with or without SeV for 12 h. **(C)** ELISA analysis of IFN-β (right) in the supernatant of Raw264.7 cells (3×10^5), transfected for 36 h with ctrl siNC or siHDAC3 (siHDAC3-1, siHDAC3-2), followed by infection with different stimulus (as shown). HDAC3-Knockdown efficiency in the Raw264.7 cells were confirmed by Quantitative RT-PCR analysis (left) and immunoblotting analysis (bottom). Data are representative of three independent experiments. Graphs show mean ± SD; n=3. * *P<0.05;* ** *P<0.01;* *** *P<0.001* (Student's t-test).

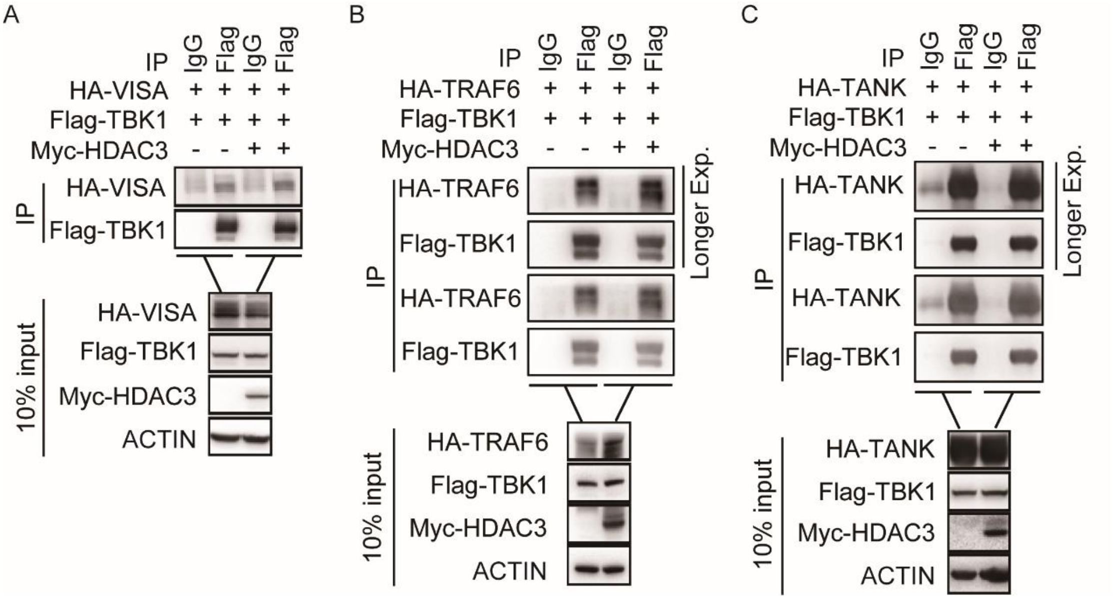

Supplementary Figure 2. Overexpression of HDAC3 has no effect on the interaction between VISA/TRAF6/TANK and TBK1. (A-C) Effect of overexpression of HDAC3 on the interaction of VISA-TBK1, TRAF6-TBK1 or TANK-TBK1. Immunoblot analysis of HEK293T cells (1.5×10^6) transiently transfected for 48 h with the indicated plasmids before co-immunoprecipitation (with anti-Flag or IgG as a control) and immunoblot analysis (with anti-HA, anti-Flag, anti-Myc or anti-ACTIN). Data are representative of three independent experiments.

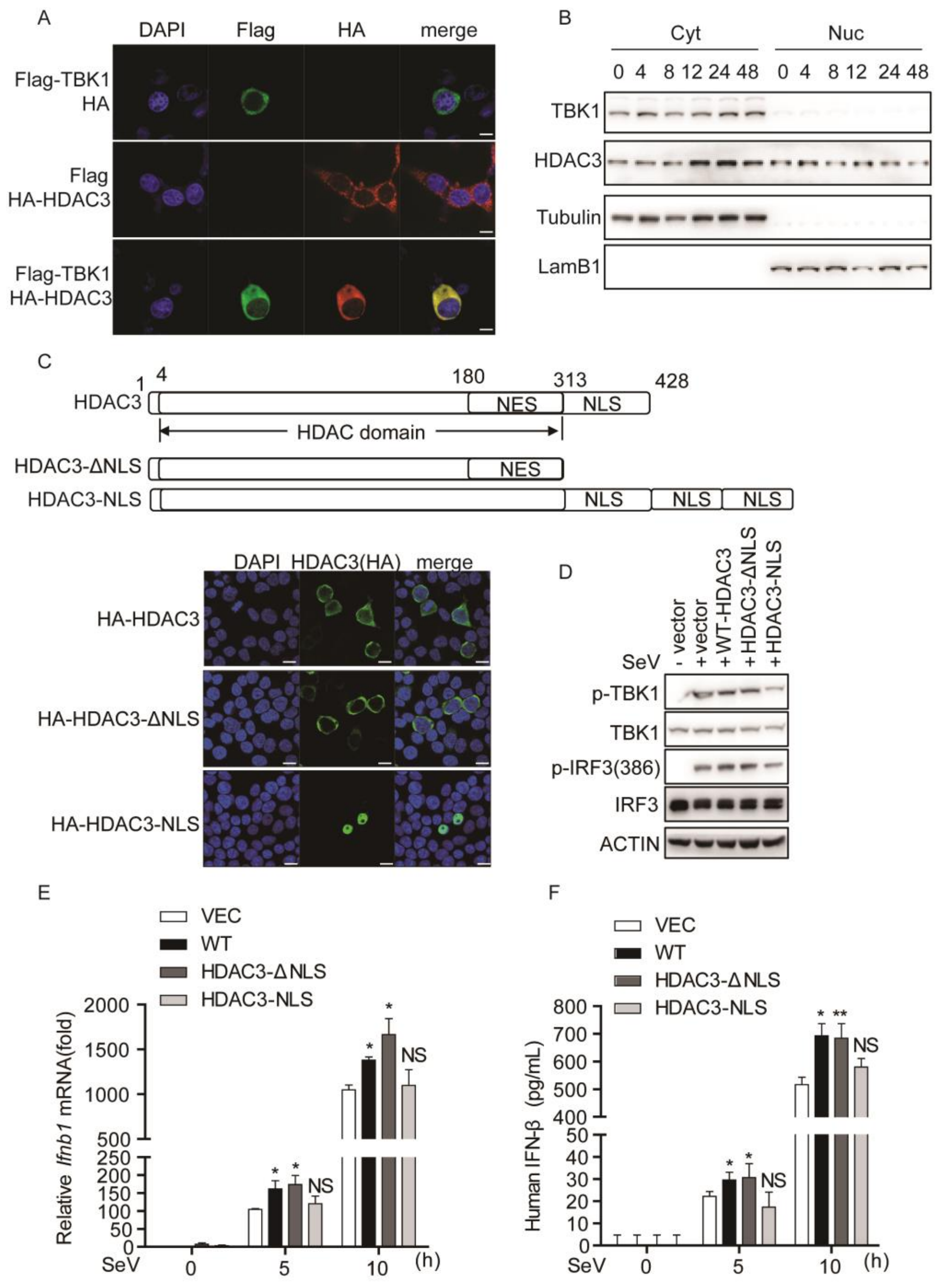

Supplementary Figure 3. The effect of HDAC3 on the IRF3 activation. (A) Confocal microscopy images of HEK293T cells (2×10^5) transiently co-transfected for 48 h with HA tagged HDAC3 or Flag tagged TBK1 plasmids as indicated (100 ng), probed with the DNA-binding dye DAPI (blue), anti-Flag (green), anti-HA (Red) Scale bar, 10 μm. **(B)** Immunoblot analysis of subcellular distribution of endogenous

HDAC3 and TBK1 proteins in HEK293T cells infected with SeV at different times post, followed by nucleus-cytoplasm extraction (5% of cytoplasmic extracts and 10% of nuclear extracts separated by SDS-PAGE). **(C)** Confocal microscopy images of HEK293T cells (2×10^5) transiently transfected for 48 h with HA tagged wild-type (WT), HDAC3 ΔNLS, or HDAC3-NLS (2×NLS HDAC3) plasmids (100 ng), probed with the DNA-binding dye DAPI (blue) and anti-HA (green) Scale bar, 10 μm. **(D)** Immunoblot analysis of phosphorylated and total TBK1 or IRF3 in HEK293T cells (2×10^5) transiently transfected for 48 h with HA tagged wild-type (WT), HDAC3 ΔNLS, or HDAC3-NLS plasmids (100 ng), followed by infection with or without SeV for 10 h. **(E&F)** Quantitative RT-PCR analysis of *IFNB1* **(E)** and ELISA of IFN-β analysis **(F)** in HEK293T cells (1×10^5) transiently transfected for 48 h with vectors (VEC), HA tagged wild-type (WT), HDAC3 ΔNLS, or HDAC3-NLS (2×NLS HDAC3) plasmids (100 ng), followed by infected with SeV for 0, 5, 10 h. Data are representative of three independent experiments. Graphs show mean ± SD; n=3. NS, no significant differences； * *P<0.05;* ** *P<0.01* (Student's t-test).

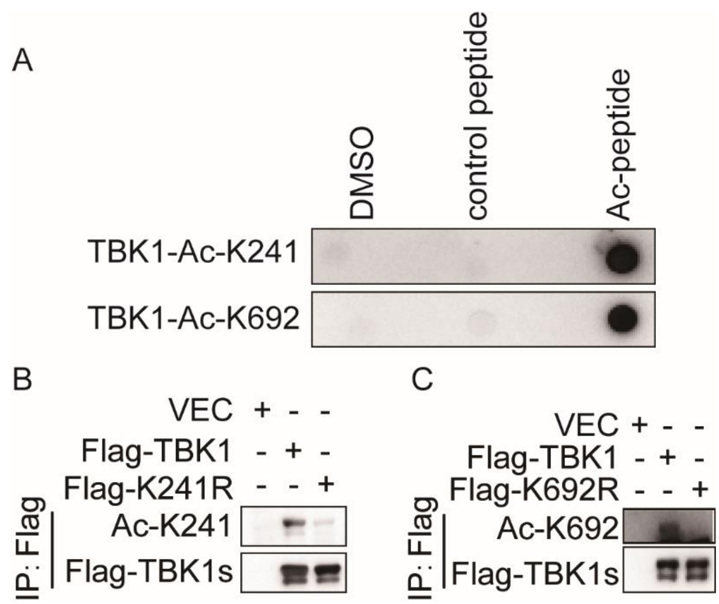

Supplementary Figure 4. Specificity of the antibody to TBK1 acetylated at Lys241 and Lys692. (A) Dot immunoblot analysis of K241 and K692 acetylation of TBK1 by using antibody specifically recognizing indicated peptide with acetylated K241 and K692 of TBK1. Dot immunoblot analysis of non-acetylated K241 and K692 of TBK1 by using antibody recognizing the indicated peptide (control peptide) is the loading control. **(B&C)** Immunoblot analysis of HEK293T cells (4×10^5) transiently transfected for 48 h with Flag-tagged wild type (WT) or TBK1 mutant K241R **(B)**, K692R **(C)** (2 μg each), immunoblot analysis were performed with the indicated antibodies.

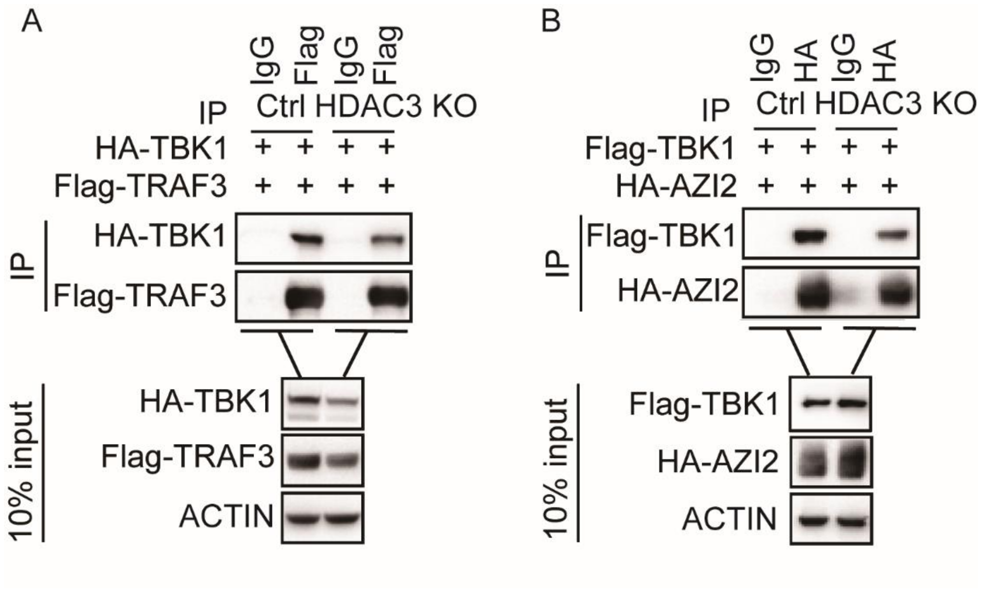

Supplementary Figure 5. Knockout of HDAC3 decreased the interaction between TRAF3/AZI2 and TBK1. (A&B) Immunoblot analysis of HDAC3-knockout cells (1.5×10^6) transiently transfected for 48 h with the indicated plasmids before co-immunoprecipitation (with anti-Flag **(A)**, anti-HA **(B)** or IgG as a control) and immunoblot analysis were performed with the indicated antibodies. Data are representative of three independent experiments.

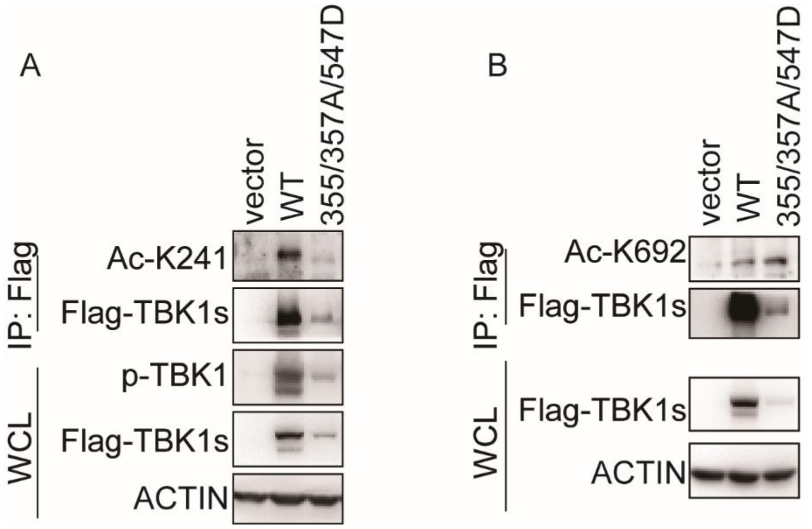

Supplementary Figure 6. The acetylation of TBK1 at K692 increased by preventing the form of TBK1 dimerization. (A&B) Immunoblot analysis of HEK293T cells (1.5×10^6) transiently transfected for 48 h with Flag-tagged empty vector, wild type (WT) or Flag-TBK1-355/357A/547D before immunoprecipitation (with anti-Flag), and immunoblot analysis with antibody to TBK1 acetylated at Lys241（Ac-K241）**(A)** and Lys692 (Ac-K692) **(B)**, anti-Flag. Data are representative of three independent experiments.

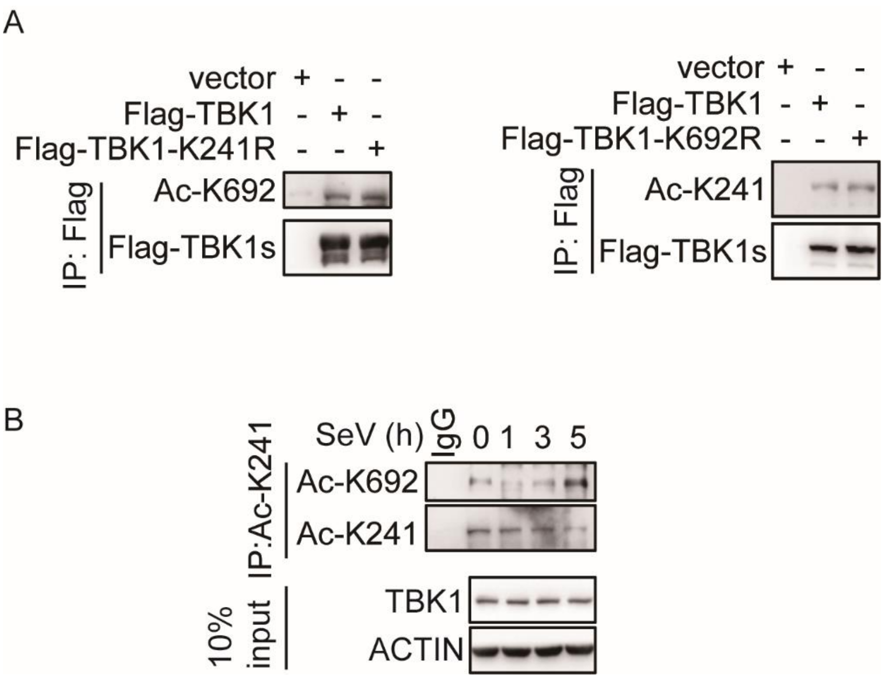

Supplementary Figure 6. The acetylation of Lys241 or Lys692 is independent each other. (A) Immunoblot analysis of HEK293T cells (4×10^5) transiently transfected for 48 h with Flag-tagged wild type (WT) or TBK1 mutant K241R (left), K692R (right) (2 μg each), immunoblot analysis were performed with the indicated antibodies. **(B)** Immunoblot analysis of endogenous Lys692 acetylation of TBK1 (K692 Ac) in HEK293T cells, followed by infection with SeV for 0-5 h, before co-immunoprecipitation (with anti-K241Ac or IgG as a control) and immunoblot analysis with anti-K692Ac, total TBK1.

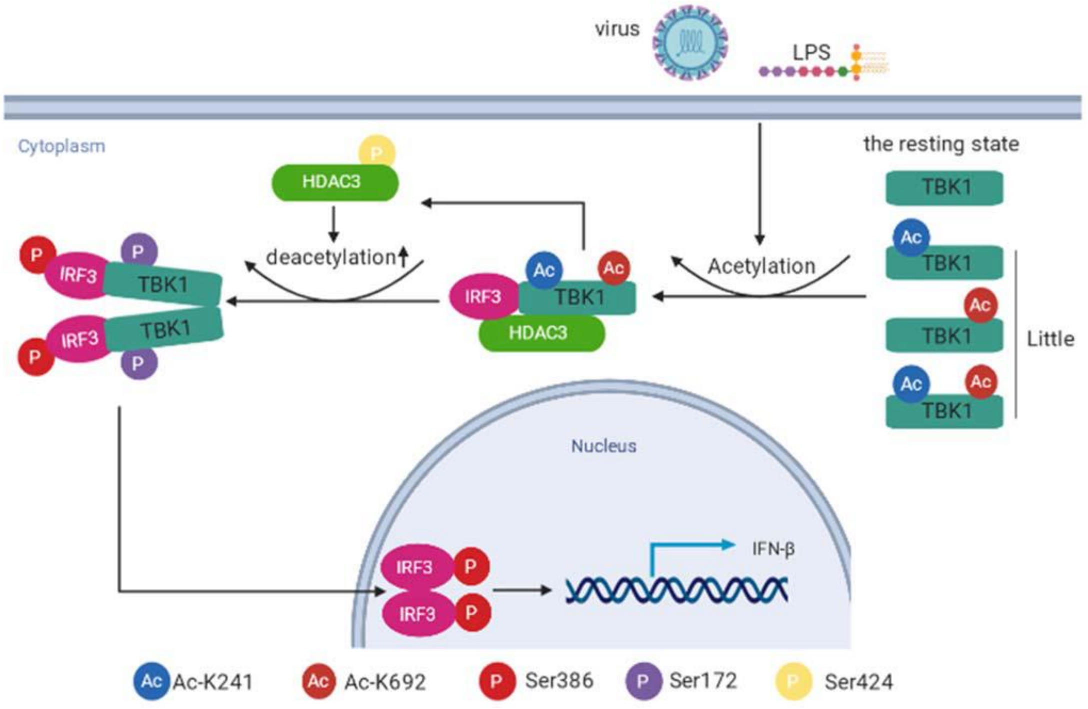

Supplementary Figure 8. The working model.

Supplementary Table 1 Antibodies used in this study.

Company	Product name	Source	Catalog No.
Cell Signaling Technology	HDAC3	Mouse	#3949S
	TBK1/NAK	Rabbit	#3013S
	Phospho-TBK1/NAK(Ser172)	Rabbit	#5483S
	Phospho-IRF3 (Ser 396)	Rabbit	#29047
	HA	Rabbit	#3724
	DYKDDDDK	Rabbit	#2368S
	Myc	Rabbit	#2272S
	GFP	Rabbit	#2555S
	Phospho-Threonine	Rabbit	#9381S
	Phospho-HDAC3(Ser424)	Rabbit	#3815S
sigma-Aldrich	Flag M2	Mouse	F1804
	HA	Mouse	H9658
Proteintech	IRF3	Rabbit	#11312-1-AP
	HDAC3	Rabbit	#10255-1-AP
	GFP	Rabbit	#50430-2-AP
	GAPDH	Mouse	#60004-1-lg
	beta Actin	Mouse	#66009-1-lg
Abcam	Phospho-IRF3 (Ser 386)	Rabbit	#ab76493
	Acetyl Lysine	Rabbit	#ab21623
	Anti-NAK/TBK1	Rabbit	#ab109735

Supplementary Table 2 Sequences of PCR primers for Expressing Vectors used in this study.

Gene	Primer	Sequences (5'-3')
HDAC3	F	CCGCTCGAGATGGCCAAGACCGTGGCCTATTTC
	R	CGGGGTACCTTAAATCTCCACATCGCTTTCCTTG
HDAC3 (1-316)	R	CGGGGTACCTTATTCTACCAGCAGCGATGTCTC
HDAC3 (317-428)	F	CCGCTCGAGATGGAGGCCATTAGTGAGGAGCTTC
HDAC3-H134Q	OCF	GGTCTGCAGCATGCCAAGAAG
	OCR	CTTCTTGGCATGCTGCAGACC
TBK1	F	ATAAGAATGCGGCCGCCATGCAGAGCACTTCTAATCATCTGTGGC
	R	CGGGGTACCCTAAAGACAGTCAACGTTGCGAAGG
TBK1-K30R	OCF	CGTGGAAGACATAAGAGAACTGGTG
	OCR	CACCAGTTCTCTTATGTCTTCCACG
TBK1-K154R	OCF	GACAGTCTGTGTACAGACTCACAGA
	OCR	TCTGTGAGTCTGTACACAGACTGTC
TBK1-K236R	OCF	GTGATGTATAGAATAATTACAGGAAAGCC
	OCR	GGCTTTCCTGTAATTATTCTATACATCAC
TBK1-K241R	OCF	CAGGAAGGCCTTCTGGTGCA
	OCR	TGCACCAGAAGGCCTTCCTG
TBK1-K251R	OCF	CTGGAGTACAGAGAGCAGAAAATGG
	OCR	CCATTTTCTGCTCTCTGTACTCCAG
TBK1-K607R	OCF	CAGATGAATGTGTTAGAAAGTATGAGGC
	OCR	GCCTCATACTTTCTAACACATTCATCTG
TBK1-K646R	OCF	GAAGAAGAAGTATCAAGATATCAAGAATATAC
	OCR	GTATATTCTTGATATCTTGATACTTCTTCTTC
TBK1-K691R	OCF	CTTGGTATGAGGAAATTAAAGGAAG
	OCR	CTTCCTTTAATTTCCTCATACCAAG
TBK1-K692R	OCF	CTCTTGGTATGAAGAGATTAAAGG
	OCR	CCTTTAATCTCTTCATACCAAGAG
TBK1-K30Q	OCF	CGTGGAAGACATAAGCAAACTGGTG
	OCR	CACCAGTTTGCTTATGTCTTCCACG
TBK1-K154Q	OCF	GACAGTCTGTGTACCAACTCACAGA
	OCR	TCTGTGAGTTGGTACACAGACTGTC
TBK1-K236Q	OCF	GTGATGTATCAAATAATTACAGGAAAGCC
	OCR	GGCTTTCCTGTAATTATTTGATACATCAC
TBK1-K241Q	OCF	CAGGACAGCCTTCTGGTGCA
	OCR	TGCACCAGAAGGCTGTCCTG
TBK1-K251Q	OCF	CTGGAGTACAGCAAGCAGAAAATGG

	OCR	CCATTTTCTGCTTGCTGTACTCCAG
TBK1-K607Q	OCF	CAGATGAATGTGTTCAAAAGTATGAGGC
	OCR	GCCTCATACTTTTGAACACATTCATCTG
TBK1-K646Q	OCF	GAAGAAGAAGTATCACAATATCAAGAATATAC
	OCR	GTATATTCTTGATATTGTGATACTTCTTCTTC
TBK1-K691Q	OCF	CTTGGTATGCAGAAATTAAAGGAAG
	OCR	CTTCCTTTAATTTCTGCATACCAAG
TBK1-K692Q	OCF	CTCTTGGTATGAAGCAATTAAAGG
	OCR	CCTTTAATTGCTTCATACCAAGAG
TBK1	OCF	CTTATCTACGCAGGGGCACGCTTAGTC
355A/357A	OCR	GACTAAGCGTGCCCCTGCGTAGATAAG
TBK1 547D	OCF	CATCCGAAAGACGACAATGTAG
	OCR	CTACATTGTCGTCTTTCGGATG
IRF3	F	CCCAAGCTTATGGGAACCCCAAAGCCACGG
	R	GGGGTACCTCAGCTCTCCCCAGGGCCCTGG
TRAF3	F	CCCAAGCTTATGGAGTCGAGTAAAAAGATGGAC
	R	CGGGGTACCTCAGGGATCGGGCAGATCCGAAGTATC
TRAF6	F	CCCAAGCTTATGAGTCTGCTAAACTGTGAAAACAGCTGTGG
	R	CGGGGTACCCTATACCCCTGCATCAGTACTTCG

F indicate forward primers; R indicate reverse primers; OCF and OCR indicate overlapping complementation forward or reverse.

Supplementary Table 3 Sequences of Q-PCR primers used in this study.

Gene name	Primer	Sequences (5'-3')
HDAC3	F	TCTGGCTTCTGCTATGTCAACG
	R	CCCGGTCAGTGAGGTAGAAAG
IFNB1	F	AGGACAGGATGAACTTTGAC
	R	TGATAGACATTAGCCAGGAG
Actin	F	GTGACGTTGACATCCGTAAAGA
	R	GCCGGACTCATCGTACTCC
VSV-G	F	CAAGTCAAAATGCCCAAGAGTCACA
	R	TTTCCTTGCATTGTTCTACAGATGG
HSV	F	ACGACAGTGGCATAGGTTGG
	R	CCGACATCACAAGGGACCTC
SeV	F	GCCAGAGGAGCACAGTCTCAGT
	R	GTCCAATGAGTGAGCTAGGAAGG
mHDAC3	F	GAAGATGCTGAACCATGCAC
	R	GGCCTGCTGTAGTTCTCCTC
mActin	F	AGTGTGACGTTGACATCCGT
	R	GCAGCTCAGTAACAGTCCGC

F indicate forward primers; R indicate reverse primers.

Supplementary Table 4 Targeting sequences of HDAC3 and TBK1 used in this study.

Primer name	Number	Sequences (5'-3')
HDAC3 KO	1#	AGTCTTAATGCCTTCAACGT
	2#	GTCAGCCCCACCAATATGCA
TBK1 KO	1#	AGAGCACTTCTAATCATCTG
siNC	1#	UUCUCCGAACGUGUCACGU
siHDAC3(Human)	1#	CCCGCAUCGAGAAUCAGAA
	2#	CAGCCGGUUAUCAACCAGGUA
siHDAC3(Mouse)	1#	CCCGCAUCGAGAAUCAGAA
	2#	GUUGAAUAUGUCAAGAGUU
shHDAC3	1#	CAAGAGTCTTAATGCCTTCAA
	2#	GATAGCTATCTGGGACATTAT
